# Prediction of the treatment effect of FLASH radiotherapy with synchrotron radiation from the Circular Electron–Positron Collider (CEPC)

**DOI:** 10.1107/S1600577524006878

**Published:** 2024-08-22

**Authors:** Junyu Zhang, Xiangyu Wu, Pengyuan Qi, Jike Wang

**Affiliations:** a Institute for Advanced Studies Wuhan University Wuhan China; b Cancer Center, Union Hospital, Tongji Medical College Huazhong University of Science and Technology Wuhan China; University of Tokyo, Japan

**Keywords:** FLASH radiotherapy, treatment effect, Circular Electron–Positron Collider, CEPC, synchrotron radiation, simulations

## Abstract

The Circular Electron–Positron Collider (CEPC) in China can also work as an excellent powerful synchrotron light source, which can generate high-quality synchrotron radiation. This synchrotron radiation has potential advantages in the medical field as it has a broad spectrum, with energies ranging from visible light to X-rays used in conventional radiotherapy, up to several megaelectronvolts. FLASH radiotherapy is one of the most advanced radiotherapy modalities. It is a radiotherapy method that uses ultra-high dose rate irradiation to achieve the treatment dose in an instant; the ultra-high dose rate used is generally greater than 40 Gy s^−1^, and this type of radiotherapy can protect normal tissues well. In this paper, the treatment effect of CEPC synchrotron radiation for FLASH radiotherapy was evaluated by simulation. First, a *Geant4* simulation was used to build a synchrotron radiation radiotherapy beamline station, and then the dose rate that the CEPC can produce was calculated. A physicochemical model of radiotherapy response kinetics was then established, and a large number of radiotherapy experimental data were comprehensively used to fit and determine the functional relationship between the treatment effect, dose rate and dose. Finally, the macroscopic treatment effect of FLASH radiotherapy was predicted using CEPC synchrotron radiation through the dose rate and the above-mentioned functional relationship. The results show that the synchrotron radiation beam from the CEPC is one of the best beams for FLASH radiotherapy.

## Abstract 2

The synchrotron radiation beam from the Circular Electron–Positron Collider (CEPC) in China can be considered as one of the best beams for FLASH radiotherapy after using a physicochemical model to predict the macroscopic therapeutic effect.

## 1. Introduction

The Circular Electron–Positron Collider (CEPC) (CEPC Study Group, 2018 ▸) is a large-scale international scientific project initiated and hosted by China. It is an electron–positron collider with a circumference of 100 km, including linear accelerators, energy intensifiers, colliders and other important components. The CEPC can also work as an excellent powerful synchrotron light source. It has electrons with energies up to 120 GeV, which is much higher than any other synchrotron light source, and it can produce better quality synchrotron radiation when these electrons move around the storage ring. The light source has a wide spectrum, from visible light to X-rays (several hundred kiloelectronvolts) used in conventional treatments, reaching up to several mega­electronvolts.

At present, FLASH radiotherapy is a hot research topic in the medical field. However, research on photon FLASH radiotherapy lags behind that of electron FLASH radiotherapy. This is because, compared with the more easily obtained electron linear accelerator (LINAC), the generation of X-rays in a photon linear accelerator is limited by electron heat deposition. The number of synchrotron radiation facilities available is relatively small (Montay-Gruel *et al.*, 2022 ▸). Therefore, photon FLASH radiotherapy equipment is also one of the research directions. Photon linear accelerators suitable for FLASH radiotherapy have been successfully designed (Liu *et al.*, 2023 ▸) and synchrotron radiation which can provide higher dose rates is also worthy of attention. The excellent properties and spectrum of the CEPC are a great help to frontier research in the medical field, so it makes sense to study the feasibility of using CEPC synchrotron radiation for advanced FLASH radiotherapy.

FLASH radiotherapy requires the delivery of pulsed rays in an extremely short time and uses ultra-high dose rate irradiation to reach the treatment dose instantly (Favaudon *et al.*, 2014 ▸; Maxim *et al.*, 2019 ▸). The ultra-high dose rate used is generally greater than 40 Gy s^−1^. One drawback of traditional radiotherapy is that the beam can cause great damage to the normal tissue around the tumor, which can affect human health. FLASH radiotherapy can protect normal tissue by a mechanism known as the FLASH effect. When this occurs, normal tissue shows reduced toxicity while tumor control is unchanged, so while killing tumor cells, normal tissue can be effectively protected to avoid adverse reactions. In addition, the movement of tumor cells due to breathing during radiotherapy can affect the accuracy of beam irradiation, reduce the effectiveness of treatment and cause greater damage to the surrounding normal tissue. FLASH radiotherapy, due to its extremely short exposure time, can greatly improve the treatment effect and avoid motion errors in the treatment process (Wang *et al.*, 2021 ▸). The advantages of FLASH radiotherapy have been found in studies of multiple tissues and organs, such as the lungs, brain, intestines and blood (Borghini *et al.*, 2024 ▸). It has even shown good treatment effects on human skin (Bourhis *et al.*, 2019 ▸). Therefore, FLASH radiotherapy is a better treatment method than conventional radiotherapy and has become a research hotspot in the field of radiotherapy.

The mechanism of FLASH radiotherapy is a hot topic in current research and many explanations have been put forward (Gao *et al.*, 2022 ▸). First, in terms of oxygen depletion, the ultra-fast delivery of single-dose FLASH radiotherapy depletes oxygen in normal tissues, thereby increasing their resistance to radiation (Montay-Gruel *et al.*, 2019 ▸; Pawelke *et al.*, 2021 ▸; Pratx & Kapp, 2019 ▸), and this at least partially explains how FLASH radiotherapy protects normal tissue from damage. For tumors, local oxygen depletion induced by FLASH radiotherapy has little impact, since their radiation response is decided by the total dose delivered. Second, a large number of reactive oxygen species can be generated in a very short time at an ultra-high dose rate, and the dense reactive oxygen species undergo self-recombination and transform into substances harmless to normal cells, resulting in the FLASH effect (Labarbe *et al.*, 2020 ▸; Hu *et al.*, 2023 ▸; Wardman, 2020 ▸). Third, for an immune response, FLASH radiotherapy will reduce the number and time of irradiation doses of immune cells and reduce the damage to the immune system (Jin *et al.*, 2020 ▸; Venkatesulu *et al.*, 2019 ▸).

Although there are many explanations, the mechanism of FLASH radiotherapy remains unclear. Therefore, it is difficult to combine all mechanisms directly to model and evaluate the therapeutic effect of FLASH radiotherapy using CEPC synchrotron radiation. Labarbe and co-workers solved the nine differential rate equations resulting from the radiolytic and enzymatic reactions network using the published values of these reaction rate constants in a cellular environment and proposed a physicochemical model of reaction kinetics to explain normal tissue sparing by FLASH radiotherapy (Labarbe *et al.*, 2020 ▸). This model built the relationship between dose rate, dose and the normal tissue complications probability (NTCP), which could be used to give a rough estimate of the treatment effect of medical beams. Therefore, we built a synchrotron radiation beamline using the *Geant4* simulation software (Agostinelli *et al.*, 2003 ▸), and the treatment effect of FLASH radiotherapy with CEPC synchrotron radiation was predicted with the model above.

## 2. Methods

### 2.1. Calculation of CEPC bending magnet source photon number

The energy of electrons in the CEPC storage ring is 120 GeV. Synchrotron radiation is produced when electrons pass through a bending magnet. The total energy emitted by a single electron per turn is 
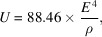
where the unit of radiated energy *U* is kiloelectronvolts, the unit of electron energy *E* is gigaelectronvolts and the unit of the radius of curvature ρ is metres.

If the current of the circulating particles is *I*, the total power of the synchrotron radiation is 
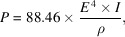
where the unit of power *P* is watts and the unit of current *I* is milliamps.

The radiated power per unit length is thus
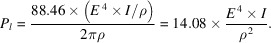


The critical energy of synchrotron radiation is used to characterize the ‘hardness’ of the radiation. The emitted radiation energies on both sides of the critical energy are equal and defined by the following expression: 
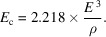
The photon spectrum produced by a single electron is 
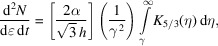
where α is the fine structure constant, *h* is Planck’s constant, γ is the ratio of total energy *E* to *m*_0_*c*^2^ and *K* is the modified Bessel function of order 5/3.

Finally, the total number of photons per second per unit length is calculated to be 2.363 × 10^16^ photons m^−1^ s^−1^.

For the CEPC, the bending angle of the bending magnet is 2.844 mrad and the radius of curvature is 10.700 km, so the number of photons emitted by the bending magnet is 7.19 × 10^17^ photons s^−1^. Therefore, we can estimate the dose rate of the CEPC medical beamline using Monte Carlo simulation to obtain the average dose produced by a single photon.

### 2.2. Simulation method

This article uses two simulation tools, *SHADOW* and *Geant4* (Version 4.10.2). The *SHADOW* software is used to generate photons emitted by the bending magnet using the Monte Carlo method (Lai & Cerrina, 1986 ▸; Sanchez del Rio *et al.*, 2011 ▸). The CEPC and bending magnet parameters, including the electron beam spot size, horizontal and vertical divergence and energy, are shown in Table 1 ▸ (CEPC Study Group, 2018 ▸). These were used as inputs for the simulation to obtain the phase space state of the photons, including position, direction, energy and polarization.

**Figure 1:**
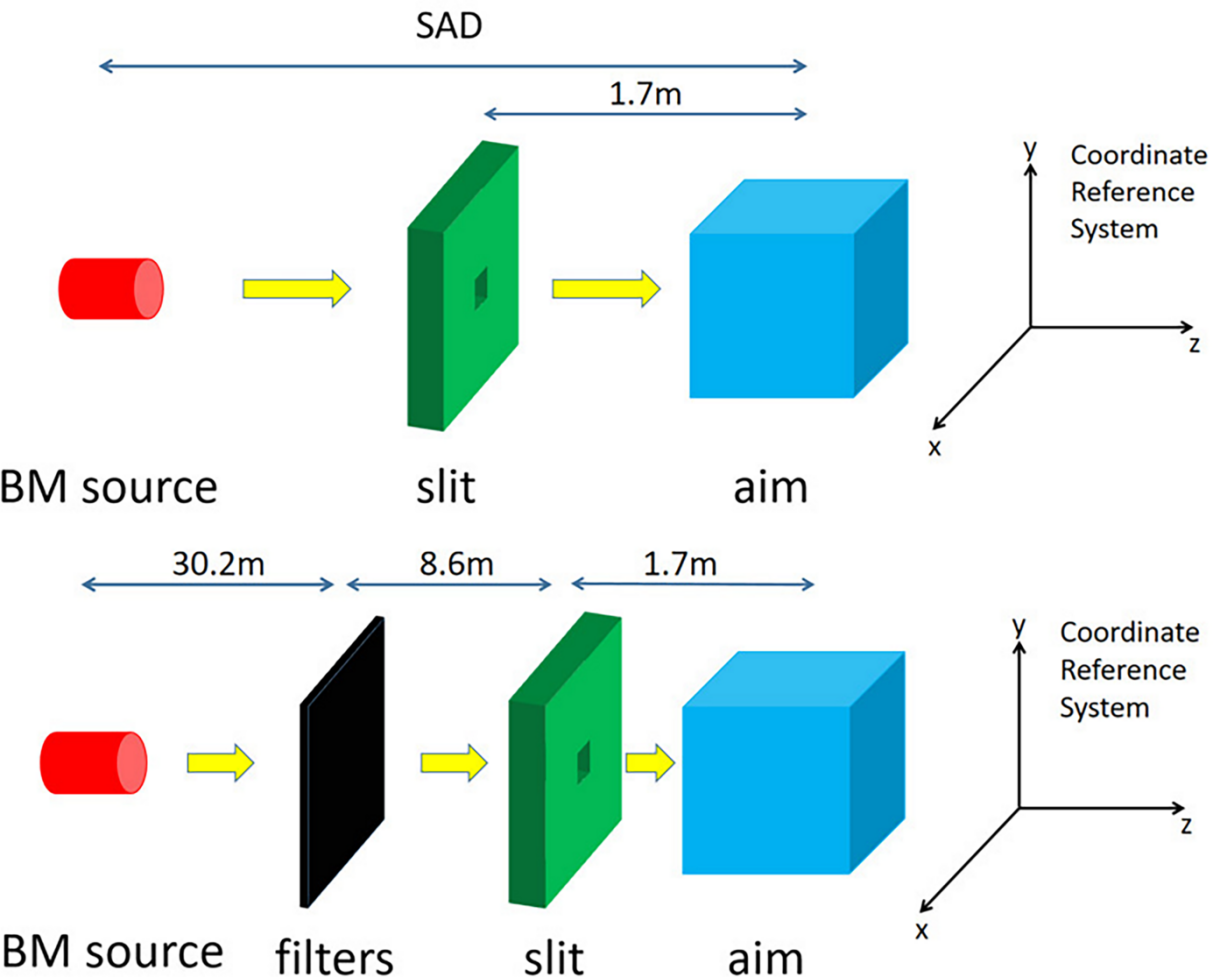
The simplified layout of CEPC beamline elements from the bending magnet to the aim position for the simulation. (Top) Without filters and (bottom) with filters. SAD is the source-to-aim distance and BM denotes the bending magnet.

The process of photon transmission from the bending magnet to the patient’s position was simulated by the Monte Carlo method using *Geant4* (Agostinelli *et al.*, 2003 ▸). The layout of the beamline (Fig. 1 ▸) is simplified without complex elements and is divided into two cases, one without low-energy filters and the other with filters. In the figure, SAD refers to the source-to-aim distance. The setting of the distance of each component from the source point is inspired by the layout of the ESRF ID17 beamline. The low-energy filters are composed of carbon, aluminium and copper foils with thicknesses of 1.42 mm, 1.52 mm and 1.04 mm, respectively, which also refer to the ones on the ESRF ID17 beamline. The patient is replaced by a water phantom which is used to measure the deposited energy of the beam. A 1 mm × 1 mm slit is added 1.7 m in front of the water phantom to limit the size of the field. Tungsten is selected as the material for the slit due to its high density, which can effectively block out-of-field photons. The slit thickness is set at 50 cm in our simulations. While this thickness is unrealistic for practical applications, it was intentionally chosen to be large enough to block all photons, thereby ensuring it is sufficient for achieving the desired outcomes in our model.

With the help of these two simulation tools, we can simply evaluate the quality of the CEPC synchrotron radiation beam and calculate the dose rate of the CEPC under this simple beamline arrangement.

### 2.3. Prediction model of FLASH radiotherapy

The occurrence of radiotherapy involves many physical and chemical reactions, such as the radiolysis of water, reactions with oxygen and radical reactions with biomolecules (Spitz *et al.*, 2019 ▸). Labarbe and co-workers solved the nine differential rate equations resulting from the radiolytic and enzymatic reactions network using the published values of these reaction rate constants in a cellular environment, and proposed a physicochemical model of reaction kinetics to explain normal tissue sparing by FLASH radiotherapy (Labarbe *et al.*, 2020 ▸). The nine differential rate equations involve nine substances including 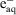, O_2_, H_2_O_2_, 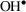, 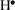, H_2_, 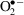, 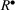 and 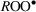, which are described in Table 2 ▸.

The peroxyl radical 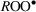 is considered to be the main cause of harmful effects on lipids and DNA, and the production of 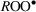 can be considered to be related to the degree of cell damage. Therefore, for semi-quantitative predictions, it is speculated that the cell biological response and the NTCP are sigmoid functions. Based on the existing experimental data, the parameters in the function are fitted.

This model establishes the relationship between dose rate, dose and treatment effect well, so we will use it to give a rough prediction of the treatment effect of FLASH radiotherapy under CEPC synchrotron radiation.

## 3. Results and discussion

### 3.1. Source characteristics

**Figure 2:**
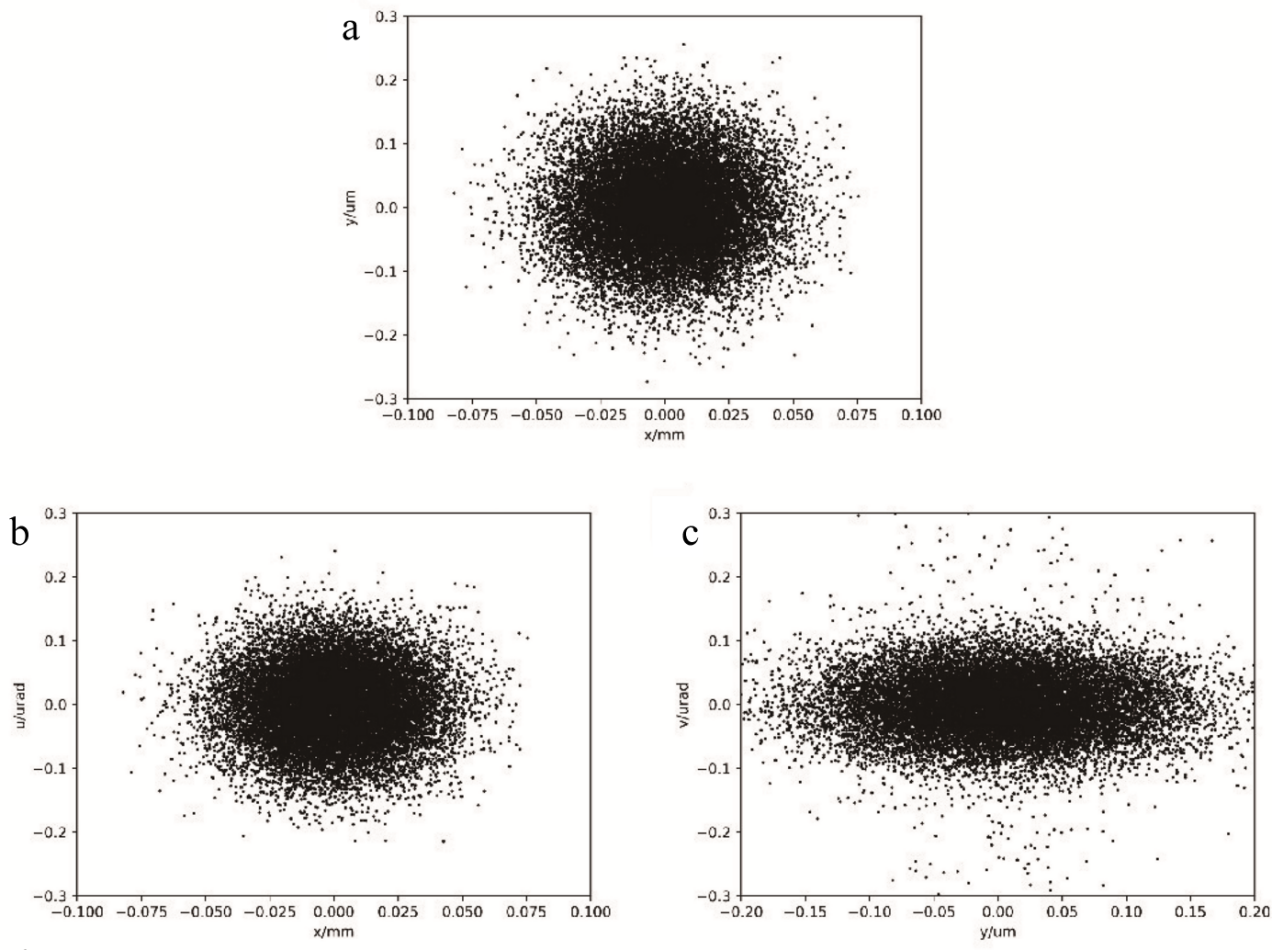
(*a*) The beam cross section at the bending magnet exit. Phase-space diagrams in (*b*) horizontal and (*c*) vertical coordinates at the end of the bending magnet. Each point represents a photon.

The photons emitted by the bending magnet were generated by the *SHADOW* software. The parameters of the CEPC and the bending magnet were input to the simulation to obtain the phase space state of the photons, including energy, position, direction and so on. The photon distribution is shown in Fig. 2 ▸, in which Fig. 2 ▸(*a*) is the cross-sectional spatial distribution of the beam, and Figs. 2 ▸(*b*) and 2 ▸(*c*) show the relationship between the position and direction of the photons. These figures show that the distribution of photons produced by *SHADOW* is axially symmetrical.

**Figure 3:**
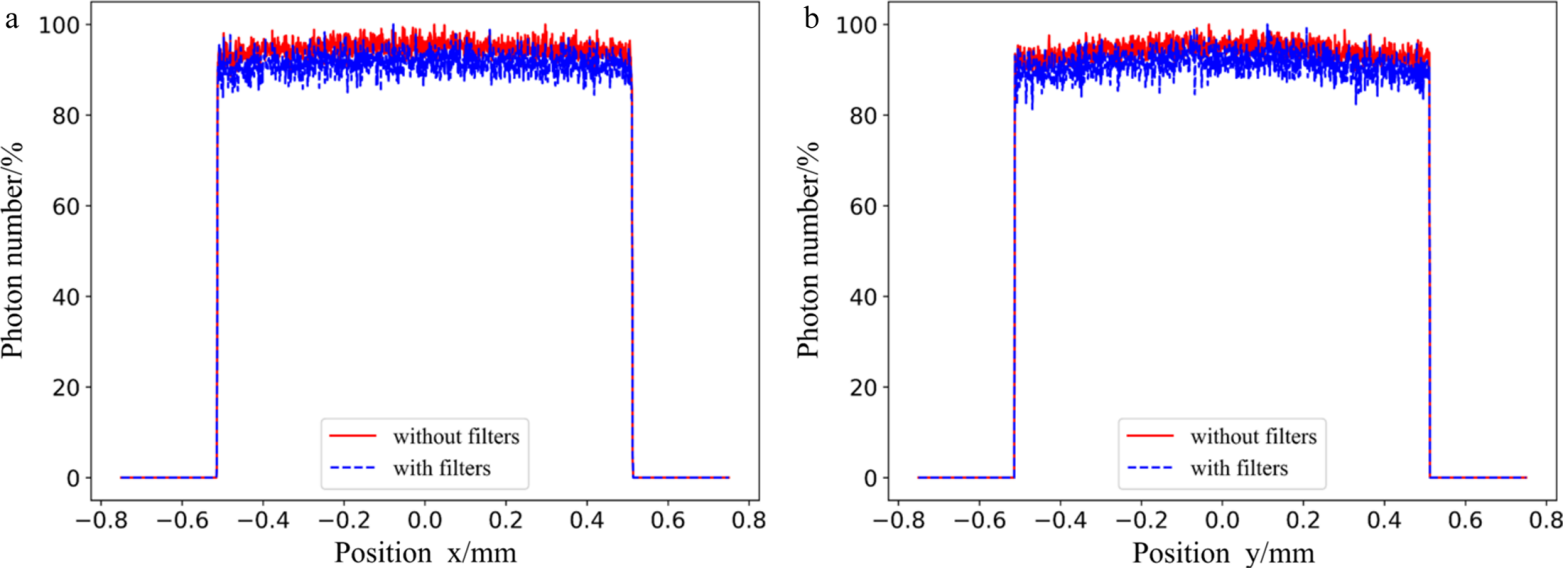
The number distribution of photons at a position 35 cm in front of the water phantom in which the maximum number of photons is normalized to 1.

The simulation results of the photon number distribution along the *x* and *y* axes are shown in Fig. 3 ▸, where the maximum number of photons is normalized to 1. The position where the photon information is collected is 35 cm in front of the water phantom. The number distribution of photons is flat and there is no large fluctuation. Without filters, the average energy of the beam is 134 keV. With filters this increases to 307 keV, which is better than 100 keV for the ESRF ID17 medical beamline. Energy affects the depth of treatment; the higher the energy, the deeper the photon enters into human tissue.

### 3.2. Dose calculation

**Figure 4:**
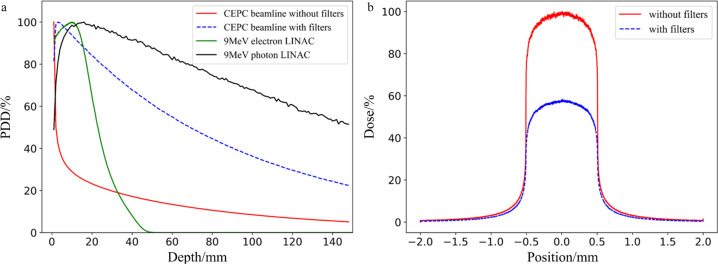
Dose distribution. (*a*) The percentage depth dose PDD with different conditions in which the maximum dose is normalized to 1. (*b*) The dose profile in the horizontal direction in which the maximum dose without filters is normalized to 1.

In radiotherapy, the percentage depth dose (PDD) is commonly used to characterize the treatment depth. The PDD relates the absorbed dose deposited by a radiation beam into a medium as it varies with depth along the axis of the beam. The dose values are divided by the maximum dose, referred to as *d*_max_, yielding a plot in terms of percentage of *d*_max_. It can be seen from Fig. 4 ▸(*a*) that the treatment depth of the beam with filters is much greater than that of the beam without filters. The PDD curves for both a 9 MeV electron beam and a 9 MeV photon beam (both produced by a general LINAC, the TrueBeam model from VARIAN) generated using *Geant4*, without filters, were simulated and compared to the CEPC beam. The result demonstrates that the CEPC beam (average energy 307 keV with filters) can offer a slightly better penetration depth than the 9 MeV electron LINAC beam. By optimizing the filter structure, the average energy of the beam can be increased and its penetration depth will be improved. While the penetration depth of a 9 MeV photon LINAC beam is superior to that of the CEPC beam, it is important to note that the dose rate from a general LINAC beam is quite low. For example, the TrueBeam medical LINAC, made by VARIAN, can deliver a dose rate of about 10 Gy min^−1^. In contrast, the CEPC synchrotron beam can deliver a very high dose rate, which is a significant advantage for certain therapeutic applications.

We selected 85% of *d*_max_ as the cut-off point, above which the effective treatment range was defined. It can be seen that the treatment depth of the beam without filters is only several millimetres, while the treatment depth of the beam with filters can reach around 2 cm, which is enough for treating superficial tumors. While the CEPC beam is currently not suitable for treating deep-seated tumors, its effective treatment depth of 2 cm is superior to other synchrotron radiation sources. Further optimizations in filter structure and increasing the average energy of the beam could potentially enhance the effective treatment depth. These kinds of optimizations will be the next focus of our work.

Dose profile is used to characterize the horizontal dose distribution, where the maximum dose without filters is normalized to 1. As can be seen from Fig. 4 ▸(*b*), the dose distribution is symmetric. Within the slit range, the doses at each position between 85% and 100% of the maximum dose are considered to be effective. This uniform distribution of the dose across the entire irradiation field means that the beam is suitable for radiotherapy.

**Figure 5:**
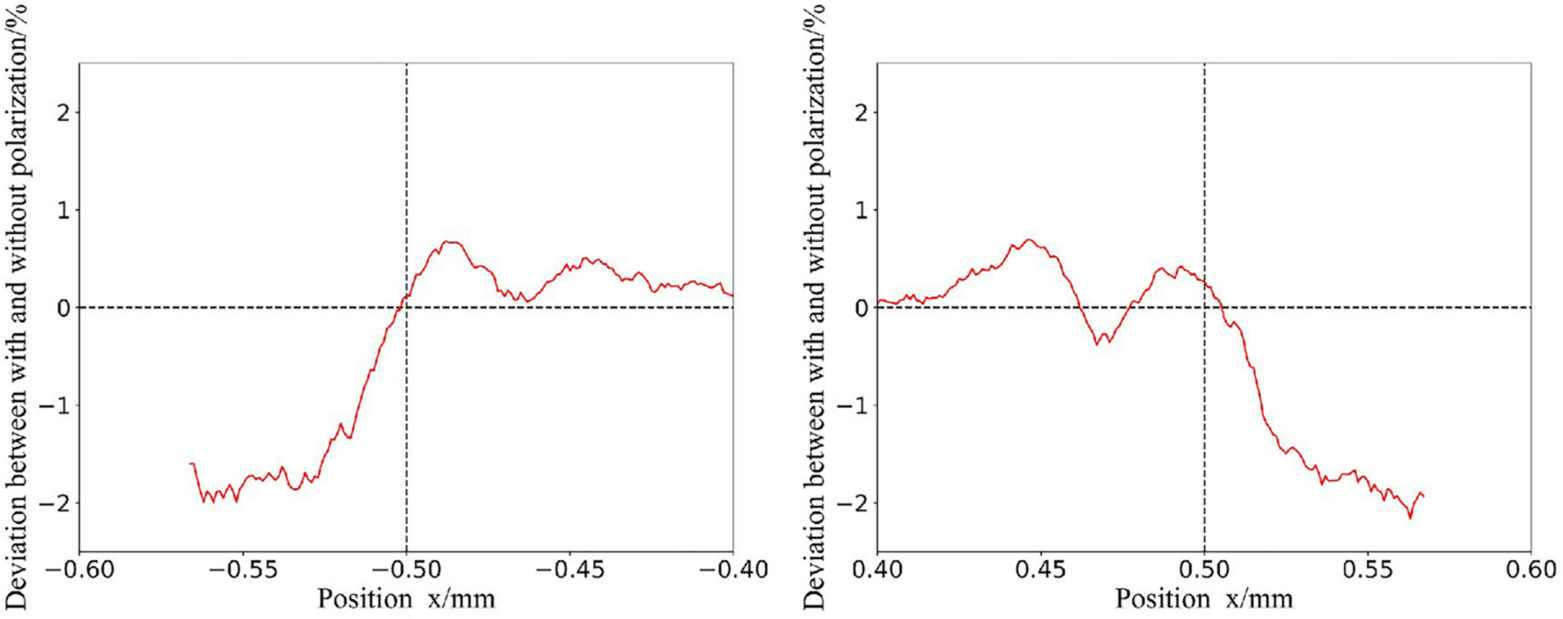
Differences at the edge of the slit between dose calculations regarding and ignoring photon polarization without filters. The *x* axis represents the horizontal position. The *y* axis represents the deviation between the polarized and non-polarized simulations.

Polarization leads to an anisotropic lack of photons compared to non-polarized isotropic scattering. This can lead to differences in the dose calculation at the slit edge with and without considering polarization. In clinical treatment, too many differences will affect the treatment plan and effect. The effect of polarization on dose calculation is also the case for the CEPC synchrotron beam. Fig. 5 ▸ shows the differences at the edge of the slit between dose calculations regarding and ignoring photon polarization without filters. The width of the slit is 1 mm, so the −0.5 mm and 0.5 mm positions in the horizontal direction are the focus of attention, and the differences are small, less than 2%. The speed of the simulation calculation can be improved by not considering the polarization. These advantages are due to the high quality of the CEPC beam.

In addition to its excellent beam quality, the CEPC has a very high dose rate, which is of great help in FLASH radiotherapy research. The dose rate *Dr* is calculated as 
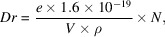
where *e* is the deposition energy of a single photon in the selected volume in electronvolts, *V* × ρ is the mass of the selected volume in kilograms and *N* is the number of photons emitted per second by the bending magnet, *N* = 7.19 × 10^17^ photons s^−1^.

Finally, through simulation and calculation, the dose rate of the simulated CEPC beamline has been obtained. Without filters, the average energy of the beam is 134 keV and the dose rate is 1.06 × 10^7^ Gy s^−1^. With filters, the average energy of the beam is 307 keV and the dose rate is 6.13 × 10^6^ Gy s^−1^. In fact, the X-rays produced by the CEPC have a pulsed structure. According to the specifications provided by the CEPC, the storage ring contains 242 bunches with a revolution frequency of 3003 Hz. This leads to approximately 726726 bunch pulses per second, with each pulse delivering about 8.44 Gy with filters, based on our earlier calculations. The period of each pulse is approximately 1.38 × 10^−6^ s and the bunch duration is roughly 14.7 ps (CEPC Study Group, 2018 ▸). Therefore, the instantaneous dose rate with filters within one pulse is about 5.74 × 10^12^ Gy s^−1^.

### 3.3. Prediction model

A physicochemical model has been developed to give a brief prediction of the treatment effect of FLASH radiotherapy using CEPC synchrotron radiation. With conventional radiotherapy, the effect of a lower dose rate on the NTCP is small and negligible. However, the influence of an ultra-high dose rate on the NTCP in FLASH radiotherapy cannot be ignored. Therefore, the physicochemical model is used to relate NTCP to dose and dose rate. In this case, we assume that the beam is a continuous photon beam.

With the mechanism of peroxyl radical 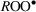 damage to lipids and DNA in normal tissues, correlating the total amount of 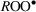 exposure in cells during radiation with the NTCP is considered. After normalization, the total amount of peroxyl radical 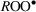
*N*(*D*, *Dr*) can be expressed by the following equation, which is determined by both dose (*D*) and dose rate (*Dr*) (Labarbe *et al.*, 2020 ▸): 
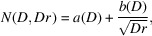
where *a*(*D*) and *b*(*D*) are fitted polynomial coefficients. By solving the nine differential rate equations, the corresponding *N*(*D*, *Dr*) of different doses and dose rates can be obtained, and the specific functional forms of *a*(*D*) and *b*(*D*) are determined by fitting: 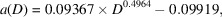

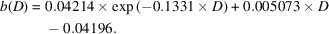
Then, for semi-quantitative predictions, the cell biological response and the NTCP can be assumed to be sigmoid functions (Labarbe *et al.*, 2020 ▸), 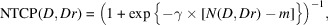
where γ and *m* are two constants that can be fitted.

**Figure 6:**
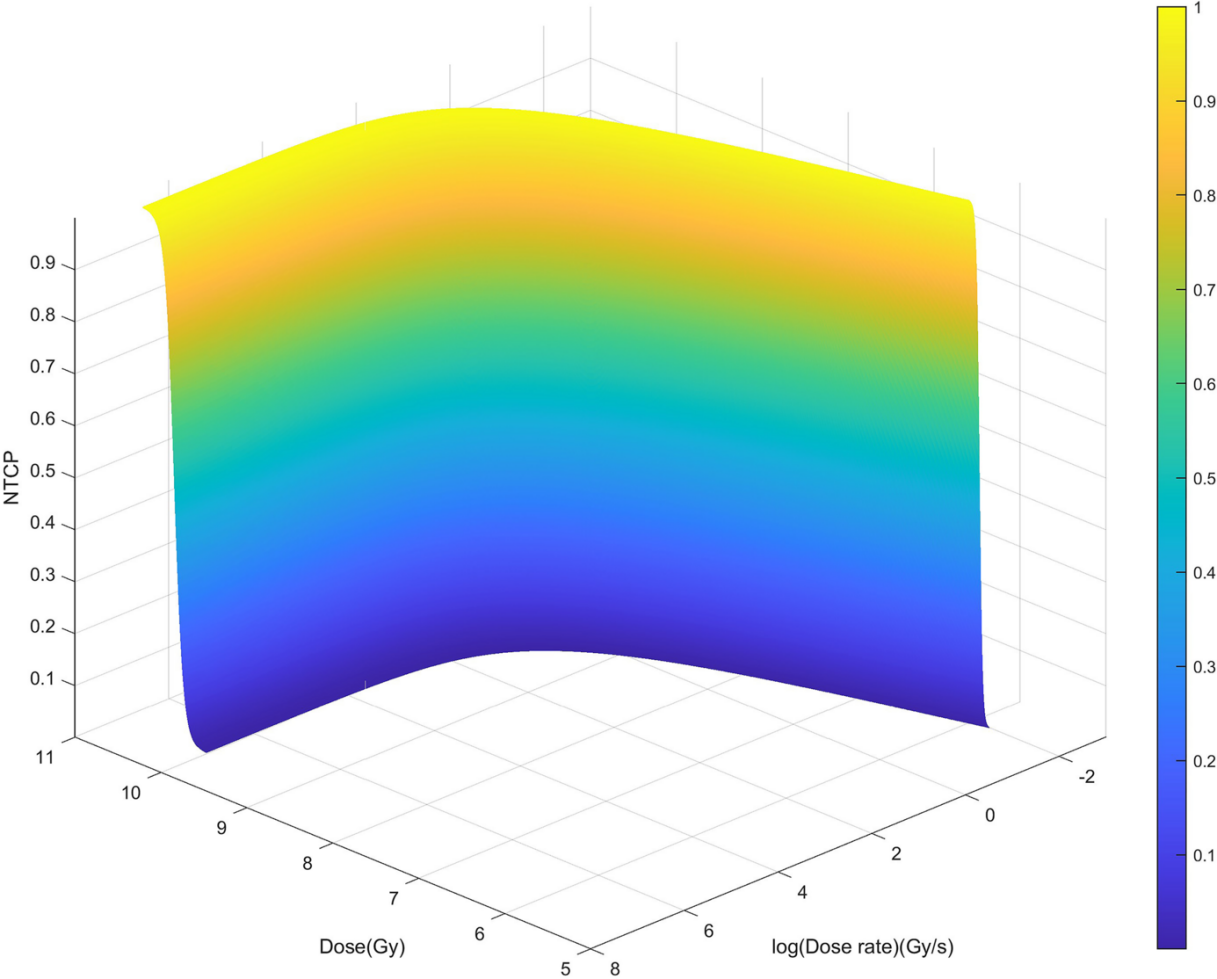
The relationship between NTCP, dose and dose rate. Within a certain range, as the dose rate increases, the tolerated dose of normal tissues also increases.

Thirteen groups of experimental data were selected from five papers (Montay-Gruel *et al.*, 2017 ▸, 2018 ▸, 2021 ▸; Alaghband *et al.*, 2020 ▸; Vozenin *et al.*, 2019 ▸) to fit the model, and the parameters γ = 1320 and *m* = 0.2002 were estimated. The side of the function image is shown in the Fig. 6 ▸. It can be seen that within a certain range, as the dose rate increases, the dose tolerated by normal tissues also increases.

**Figure 7:**
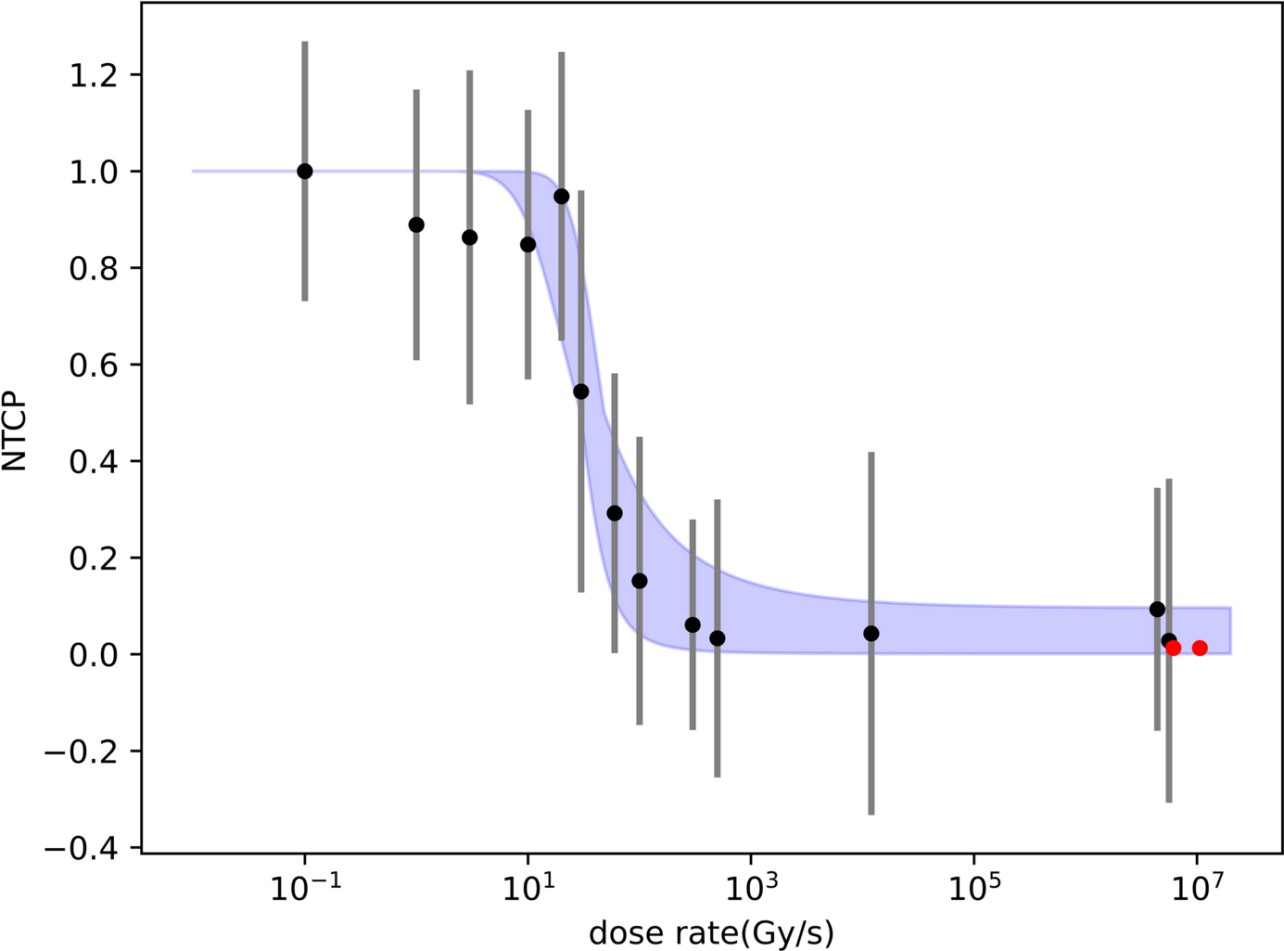
Prediction of the treatment effect with the CEPC medical beamline. Black dots represent published experimental data, while the left-hand red dot represents the simulation result for the CEPC with filters and the right-hand red dot represents the simulation result for the CEPC without filters.

For a dose of 10 Gy, the relationship between NTCP and dose rate is shown in Fig. 7 ▸. The 13 black dots represent the published experimental data, while the left-hand red dot represents the simulation result for the CEPC with filters and the right-hand red dot represents the simulation result for the CEPC without filters. The blue shaded area indicates the error margin of the fit. The *x* axis is the dose rate and the *y* axis is the NTCP. As can be seen from the figure, FLASH radiotherapy using the medical beamline of the CEPC can achieve a good treatment effect.

In our current model, we approximated the beam as continuous rather than considering its pulsed structure. While the work of Manuel Udías and co-workers considers the impact of the pulse structure, their findings indicate that the effect of pulsing is minor compared to a continuous photon beam. For example, the free radical 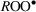 production of a pulsed photon beam is reduced by about 3% compared to a continuous photon beam (Espinosa-Rodriguez *et al.*, 2022 ▸). Therefore, we did not incorporate the pulse structure into our current work. However, we plan to refine the beamline design in future studies, gain precise control of the dose per pulse and incorporate the bunch structure into our radio-kinetic model calculations.

## 4. Conclusions

The CEPC can generate high-quality synchrotron radiation as a powerful and excellent synchrotron light source, which has great advantages in the medical field. Using the *SHADOW* and *Geant4* software, we successfully built a sample medical beamline for the CEPC, simulated the characteristics of the beam emitted by the CEPC synchrotron radiation source, and calculated the average energy and the dose rate of the beam. Without filters, the average energy of the beam is 134 keV and the dose rate is 1.06 × 10^7^ Gy s^−1^. With filters, the average energy of the beam is 307 keV and the dose rate is 6.13 × 10^6^ Gy s^−1^.

Then, referring to the physicochemical model of reaction kinetics published by Labarbe *et al.* (2020 ▸), the functional relationship between treatment effect, dose and dose rate was determined by fitting experimental data for radiotherapy.

Finally, the model was used to predict the treatment effect of FLASH radiotherapy with synchrotron radiation from the CEPC. The results show that CEPC synchrotron radiation is one of the most promising beams for FLASH radiotherapy.

## Figures and Tables

**Table 1:** CEPC and bending magnet parameters

Parameter	Value	Unit
Energy	120	GeV
σ_*x*_	2.09 × 10^−2^	mm
σ_*y*_	6.8 × 10^−5^	mm
ɛ_*x*_	1.21	nm rad
ɛ_*y*_	3.1 × 10^−3^	nm rad
Radius of curvature	10700	m

**Table 2:** Description of nine substances taken into account in the FLASH radiotherapy model

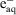	Electrons in aqueous solution, generated from water radiolysis
O_2_	Molecular oxygen, naturally present in tissues
H_2_O_2_	Hydrogen peroxide, produced further from radiolysis products interacting with oxygen
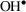	Hydroxyl radicals, a product of water radiolysis
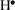	Hydrogen atoms, a product of water radiolysis
H_2_	Molecular hydrogen, formed from water radiolysis
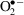	Superoxide anion, formed from the reaction of radiolysis products with oxygen
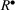	Free radicals derived from the radiolytic decomposition of carbon-based biomolecules (*R*H)
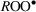	Peroxyl radicals, harmful to lipids and DNA, formed from the combination of 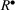 and oxygen
